# *Terminalia chebula* Retz. As a resistance-modifying botanical drug against priority pathogens: a systematic review

**DOI:** 10.3389/fphar.2026.1855899

**Published:** 2026-06-09

**Authors:** Kamran Zaman, Surthi Ravedar, Nidhi R, Kalesh Karun, Jainabbi Patel, Flemin Felix, Kranthi Kiran Akula, Nidhi Hiremath, Shivani Tendulkar, Tejaswini Salunkhe, Asif Kavathekar, Jyothi Bhat

**Affiliations:** Indian Council of Medical Research - National Institute of Traditional Medicine, Belagavi (ICMR-NITM Belagavi), Belagavi, Karnataka, India

**Keywords:** antimicrobial resistance, botanical drug, ESKAPE pathogens, metabolites, priority pathogen, resistance-modifying agents

## Abstract

**Background:**

The global rise of antimicrobial resistance (AMR) among priority bacterial pathogens represents a critical threat to public health, necessitating the development of alternative therapeutic strategies. *Terminalia chebula* Retz. (*Terminalia chebula*), a traditionally used medicinal plant rich in hydrolysable tannins, flavonoids, and polyphenolic metabolites, has emerged as a promising candidate due to its broad-spectrum antimicrobial and pharmacological properties.

**Methods:**

This systematic review critically evaluates the *in-vitro* antimicrobial activity and resistance-modifying potential of *T. chebula* fruit extracts against ESKAPE pathogens, including *Enterococcus faecium, Staphylococcus aureus, Klebsiella pneumoniae, Acinetobacter baumannii, Pseudomonas aeruginosa, Enterobacter* species and *Escherichia coli* as a representative of the Enterobacteriaceae (now Enterobacterales) family. A comprehensive literature search was conducted across PubMed, Scopus, ScienceDirect, and Google Scholar databases in accordance with PRISMA guidelines and registered in PROSPERO (CRD420251024476). Based on predefined criteria, 24 studies were included for qualitative analysis.

**Results:**

The findings demonstrate that *T. chebula* extracts exhibit *in vitro* antimicrobial activity, with ethanolic and methanolic extracts showing enhanced higher efficacy, particularly against multidrug-resistant strains of *S. aureus*, and *P. aeruginosa*, and *E. coli*. Notably, few studies reported synergistic interactions between phytochemicals and conventional antibiotics, suggesting antibiotic resistance-modifying roles. Mechanistic insights suggest that metabolites such as chebulagic acid, chebulinic acid, and gallic acid contribute to antimicrobial activity by disrupting the bacterial cell membranes, inhibiting biofilm formation, and interfering with resistance pathways. However, substantial variations in extraction methods and experimental designs limit cross-study comparability, highlighting the need for standardized protocols.

**Conclusion:**

Overall, the evidence supports the potential of *T. chebula act* as a resistance-modifying and an antibiotic adjuvant. Future studies should prioritize bioassay-guided isolation, mechanistic validation, and preclinical evaluation to facilitate translational application in combating AMR.

## Introduction

Antimicrobial resistance (AMR) has emerged as a major global public health crisis, undermining the effectiveness of existing antibiotic therapies and significantly increasing morbidity and mortality worldwide. Recent estimates indicate that bacterial AMR was associated with approximately 4.71 million fatalities, with 1.14 million deaths directly attributed to antimicrobial resistance infections. The projections suggest a substantial rise in attributable mortality by 2050 if effective interventions are not implemented. The rapid emergence and dissemination of resistant pathogens are driven by factors such as the overuse and misuse of antibiotics, horizontal gene transfer, and the ability of microorganisms to form biofilms and develop adaptive resistance mechanisms (Naghavi et al., 2024).

Among the most critical threats are the priority pathogens like ESKAPE pathogens (*Enterococcus faecium, Staphylococcus aureus, Klebsiella pneumoniae, Acinetobacter baumannii, Pseudomonas aeruginosa, Enterobacter* species) and *E. coli* (Naghavi et al., 2024; Kharat et al., 2024; World Health Organization, 2024) that are responsible for a significant proportion of healthcare-associated infections and exhibit multidrug resistance to conventional antibiotics (World Health Organization, 2024). The ability of these pathogens to bypass the effects of conventional antimicrobial therapies is a critical concern. Antibiotic resistance is mainly attributed to intrinsic adaptive capabilities of bacteria, and this process is significantly enhanced by the misuse and overuse of antimicrobial agents (Ventola, 2015; Arias and Murray, 2009). Furthermore, many pathogens can form biofilms, displaying phenotypic tolerance, which is also a cause for concern, as both factors are known to hinder drug penetration and allow pathogens to survive in the presence of high concentrations of antibiotics (Stewart and Costerton, 2001; Grooters et al., 2024). Another key factor contributing to the emergence and spread of resistance is horizontal gene transfer, a process by which pathogens acquire resistance genes via mobile genetic elements such as plasmids, thereby enabling the rapid transfer of resistance genes between bacterial species (Ghal et al., 2022; Hall and Mah, 2017). The presence of diverse and sophisticated resistance mechanisms in pathogens is the key factor enabling ESKAPE pathogens to bypass the effects of antimicrobial agents and leads to clinical consequences of failure, i.e., mortality, thereby establishing a cause-and-effect relationship between the biology of the pathogens and their clinical impact on human populations. Thus, the detailed problem statement in this regard is such that the search for novel antimicrobials, especially from under-explored sources such as plants, is not only a scientific imperative but also a strategic imperative to prevent a global catastrophe in terms of human health and economic consequences (Abreu et al., 2012; Atanasov et al., 2021; Savoia, 2012).

**FIGURE 1:**
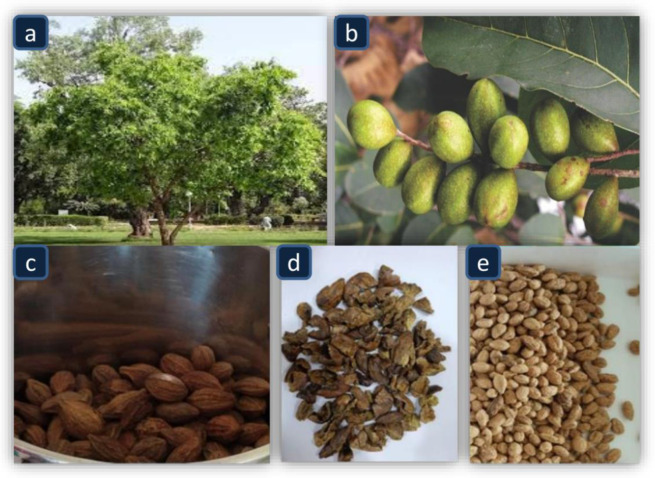
Morphological features of *Terminalia chebula* Retz.: **(a)** tree and **(b)** fresh fruits reproduced/adapted from cited sources with appropriate attribution (Baradari Gardens Patiala, 2026; Ayurveda, 2019; Mart, 2026); **(c)** dried fruits, **(d)** pericarp, and **(e)** seeds are original photographs captured by the authors.

*Terminalia chebula* Retz. (*Terminalia chebula*), commonly named black myrobalan or haritaki, is well documented medicinal plant extensively used traditional systems such as Ayurveda and Unani medicine (Walia and Arora, 2013). It is rich in bioactive chemical constituents including hydrolysable tannins, chebulic acid, gallic acid, phenolics, flavonoids, and Triterpenoids (Figure 1) In the traditional Ayurvedic literature, *T. chebula* is described as having seven different varieties, including Vijaya and Rohini, which are related to the medicinal properties and usage (Bag et al., 2013; Sharma et al., 2019). The plant has the adaptability to grow in different ecological conditions and can be found in soils with different compositions and climates, with an annual rainfall between 100–150 cm and temperatures between 1 °C and 48 °C (Prajapati et al., 2020; Walia and Arora, 2013).

The chemical composition of *T. chebula* is interesting due to the significant quantity of bioactive metabolites that it contains. The main chemical constituents include hydrolysable tannins, which make up about one-third of the total chemical composition of the various bioactive metabolites found in the plant (Bulbul et al., 2022). The main chemical constituents include phenolic acids such as gallic acid, ellagic acid, and chebulic acid; gallotannins such as 1,6-di-O-galloyl-β-D-glucose; and ellagitannins such as punicalagin, casuarinin, and corilagin (Rashed, 2024). The plant also contains chebulanin, chebulagic acid, and chebulinic acid, which have been studied extensively with respect to their pharmacological properties. The plant is also rich in flavanol glycosides, triterpenoids, and coumarin derivatives such as chebulin, which all contribute to the pharmacological value of the plant. From the comparative chemical composition, it is seen that the fruits contain high amounts of total phenolics and flavonoids such as quercetin and catechin, as well as ascorbic acid, which increases the value and pharmacological importance of the plant as a medicinal plant (Wang et al., 2024). Apart from the fruits, the seeds and galls of *T. chebula* contain oleanane-type triterpene acids as well as phenolic metabolites, which add to the pharmacological value of the plant. The plant has been studied extensively due to its varied remedial properties.

Some of the bioactive constituents of the plant include tannins like casuarinin, chebulanin, and chebulinic acid, which have been reported to have significant antioxidant properties through the inhibition of lipid peroxidation and reduction of oxidative stress-mediated cellular damage (Suchalatha and Devi, 2005; Naik et al., 2004). *Terminalia chebula* has been reported to have significant anti-inflammatory and anti-arthritic properties, mainly through the action of constituents like chebulagic acid and corilagin, which have been reported to downregulate inflammatory responses and alleviate arthritis (Ekambaram et al., 2022). The antidiabetic properties of the plant have been reported to be mediated through the inhibition of alpha-glucosidase activity, induction of insulin secretion, and reduction of blood glucose levels (Gao et al., 2007). In addition, the methanolic extracts of the plant have been reported to have significant cytotoxic effects on several types of cancer cells, including prostate, breast, and osteosarcoma, with chebulinic and chebulagic acids reported to be the main bioactive constituents responsible for the anti-cancer properties of the plant (Saleem et al., 2002).

Antimicrobial activity of *T. chebula* is also marked, particularly against multidrug-resistant strains of bacteria such as *Staphylococcus aureus, Pseudomonas aeruginosa,* and *Klebsiella pneumoniae*. Various studies have confirmed that the inhibition zones of *T*. *chebula* extract at higher concentrations are comparable to commonly used antibiotics (Kannan et al., 2009; Bag et al., 2012). The antimicrobial activity of *T. chebula* is thought to be primarily due to its high content of hydrolysable tannins, phenolic acids, and flavonoids, which are thought to interfere with microbial cell wall integrity and impede vital enzymatic activity, particularly chebulagic acid, gallic acid, and ellagic acid (Tiwana et al., 2024). Even though its antifungal activity is relatively low, the alcoholic extract of *T. chebula* exhibits activity against fungal strains such as *Candida albicans* and *Aspergillus flavus* (Rekha et al., 2014). The consistency of antimicrobial activity of *T. chebula* in different studies highlights its promising status as a drug of natural origin for antimicrobial activity, particularly in the face of increasing antimicrobial resistance. Despite the growing body of evidence, there remains a lack of comprehensive evidence, specifically *T. chebula* extract’s activity against ESKAPE pathogens and the influence of methodological variables such as extraction techniques and solvent systems on antimicrobial efficacy. Therefore, this systematic review aims to critically evaluate the *in-vitro* antimicrobial activity of *T. chebula* fruit extracts against ESKAPE pathogens, with particular emphasis on phytochemical composition, extraction methodologies, resistance-modifying potential, and implications for future pharmacological development.

## Materials and methods

### Study design and registration

This systematic review was conducted in accordance with the Preferred Reporting Items for Systematic Reviews and Meta-Analyses (PRISMA) guidelines. The study protocol was prospectively registered in the PROSPERO database under the registration number CRD420251024476, ensuring transparency and methodological rigor.

### Search strategy

A comprehensive and systematic literature search was performed across multiple electronic databases, including Scopus, PubMed, Science Direct, and Google Scholar, to identify relevant studies evaluating the antimicrobial activity of *T. chebula.* The search was conducted from the inception of the databases up to the calendar year 2025,without any restrictions on publication year. A combination of controlled vocabulary and free text terms was used. The search strategy included the following Boolean expression: ((“*E. coli” OR “Escherichia coli” OR ″P. aeruginosa” OR “P. aeruginosa” OR “K. pneumoniae” OR “K. pneumoniae” OR ″A. baumannii” OR “Acinetobacter baumannii” OR “Priority pathogen” OR ESKAPE)* AND (“*Terminalia chebula* Retz.” OR “*T. chebula*” OR “Haritaki” OR “*T. chebula* fruit extract”) AND (“Antimicrobial activity” OR “Antimicrobial Potential” OR “Antibacterial activity” OR “Antibacterial potential” OR “Antimicrobial resistance” OR “AMR” OR “Antibiotic resistance”)) (Supplementary Table S1) Additional manual screening of the reference lists of relevant articles was performed to identify studies that may not have been captured during the database search.

### Study selection

All the retrieved records were imported Rayyan software for systematic screening and duplicate removal. Two independent reviewers screened titles and abstracts based on predefined eligibility criteria. Full-text articles were then assessed for inclusion.

### Inclusion criteria

Studies were included if they.

- Investigated antimicrobial activity of *T. chebula* extracts (primarily fruit extracts)
- Focused on ESKAPE pathogens and *E. coli* or clinically relevant resistant bacterial strains reported quantitative antimicrobial activity outcomes such as MIC, MBC, or zone of inhibitions
- *In vitro* experimental studies

### Exclusion criteria

Studies were excluded if they.

- Were review articles, conference abstracts, or lacked full-text availability
- Did not provide sufficient methodological or quantitative data
- Focused exclusively on non-bacterial pathogens (Fungi/viruses)
- Investigated plant parts or formulations without clear relevance to *T. chebula* fruit extracts

### Data extraction

A structured form was employed to extract relevant details from all the full-text research articles we had identified for this study. Each of the identified articles from the database search was downloaded in its entirety for thorough scrutiny. The data was collated in Microsoft Excel with the following headers: Sr No., Study ID, Plant Part Used, Collection Place, Extraction Method, Solvent Used, Organism Used, Experimental Methods, Number of Replicates, Antibiotic Used, and Study Outcome. The results were measured by Minimum Inhibitory Concentration (MIC mg/mL), Minimum Bactericidal Concentration (MBC mg/mL), Disc Diffusion, and Well Diffusion assays.

### Quality assessment and risk of bias

A formal risk of bias or methodological quality assessment was performed using ToxR tool for the included *in vitro* research. This tool evaluates studies in five categories: test substance characterization, test system description, study design, results reporting, and data plausibility. Each criterion was rated as 1 (adequately reported) or 0 (not reported/inadequate), with a maximum total score of 18 (Schneider et al., 2009).

### Statistical analysis

Data extracted from the various studies was neatly organized and tracked in Microsoft Excel, which helped in the initial sorting and categorization of various parameters of the study. In the statistical part of the study, SPSS was used to calculate the mean and standard deviation, which helped in a quantitative comparison of the results, such as MIC, MBC, disc diffusion, and well diffusion, of various studies included in the review. The results were then segregated based on the extraction solvents, bacterial classification, and type of pathogen. Due to the substantial heterogeneity in study design, extraction methods, microbial strains, and outcome reporting, a formal meta-analysis was not feasible. Therefore, statistical analysis was limited to descriptive measures (mean and standard deviation), and the results should be interpreted cautiously without implying pooled effect estimates.

## Results

### Study selection

**FIGURE 2:**
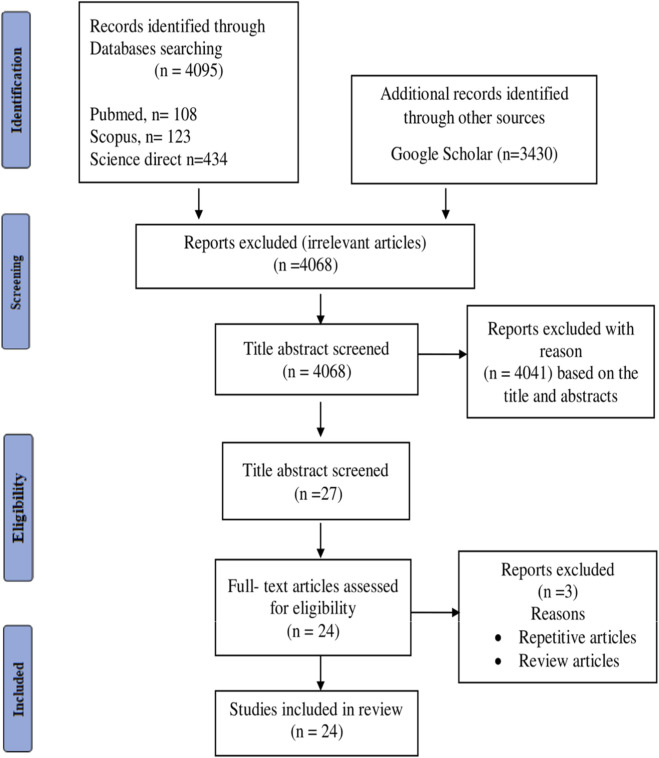
PRISMA flowchart of study selection process.

A wide search resulted in the retrieval of 4,095 articles from the electronic databases (PubMed 108, Scopus 123, Science Direct 434), and an additional 3,430 were found in Google Scholar. After screening the titles and abstracts to exclude the irrelevant ones (4,068), 27 articles were selected for detailed evaluation. Of these, three articles were excluded according to predefined exclusion criteria, which included duplicate publications and review articles. The remaining 24 full-text articles were assessed for eligibility, all of which fulfilled the inclusion criteria and were subsequently included in the final qualitative synthesis (Figure 2).

### Study characteristics

The included studies were conducted across diverse geographical regions, with the majority originating from the Indian subcontinent with no. of articles (18, 83.3%), followed by other Asian countries (n = 2, 8.3%), Africa (n = 1, 4.2%), and Australia (n = 1, 4.2%). The included studies were published between 2003 and 2024. Of the dated studies (n = 19), 73.7% (14/19) were published after 2010, indicating sustained research interest in the topic (Supplementary Table S2).

The antimicrobial activity of plant materials showed significant variation from one study to another. Fruits dominated by far (n = 20, 83.3%), followed by leaves (n = 3, 12.5%), while other plant materials, such as seeds, bark, and whole plants, each contributed one study. As for the extraction techniques used, maceration was clearly the favorite (n = 18, 75%), followed by Soxhlet (n = 6, 25%). Ethanol, methanol, and water were clearly the solvents of choice, mainly because of their polarity, or in combination in a hydroalcoholic solution (Supplementary Table S2) shows a summary of the main characteristics of all studies reviewed.

Various methods were used in the evaluation of the antimicrobial effectiveness. The most used method was the well diffusion assay, which was used in 62.5% of the studies (15 studies), followed by the disc diffusion method and the broth micro dilution method, each used in 41.7% of the studies (10 studies), although the broth micro dilution method includes the tube method, the macro method, and the micro method (Supplementary Table S2).

Well Diffusion Assay (or Agar Well Diffusion) was used in studies examining ethanol extracts, hydroalcoholic extracts, aqueous extracts, and methanolic extracts. The disc diffusion assay was used mainly to screen crude extracts and fractions, as well as to determine resistance. A select few of studies (n = 4) assessed the effects of antibiotic combinations utilizing advanced interaction-based methods. True synergistic interactions were evaluated in two investigations utilizing checkerboard titration and time-kill assays on fruit extracts in combination with standard antibiotics (gentamicin and trimethoprim). In other research, fractional inhibitory concentration (FIC) analysis was utilized to establish if extracts and antibiotics had synergistic or additive interactions (Supplementary Table S5). The remaining investigations indicated increased or potentiated antibacterial activity in the presence of antibiotics without a formal synergy measure.

The majority of studies (n = 20, 83.3%) included appropriate positive controls using standard antibiotics. A wide range of standard antibiotics were used as positive controls, including Ciprofloxacin, Gentamicin, Amoxicillin, Ampicillin, Streptomycin, Amikacin, Tetracycline, and Carbapenems (Imipenem/Meropenem) (Supplementary Table S2).

The investigated bacterial spectrum encompassed isolates representing both Gram-positive and Gram-negative pathogens. In addition to core ESKAPE pathogens, several studies also evaluated *Escherichia coli*, which was included as a representative Enterobacteriaceae organism due to its clinical importance. *Escherichia coli* was the most frequently tested organism, followed by *Staphylococcus aureus*, *E. coli* was the most frequently tested organism (n = 20 studies, 83.3%) in the studies, including those focusing on Uropathogenic *E. coli* (UPEC) and general enteric pathogens. *Staphylococcus aureus* was evaluated (n = 17 studies, 70.8%), including standard ATCC strains and clinical MDR isolates *P. aeruginosa* was tested (n = 10 studies, 41.7%), especially due to its resistance profile in nosocomial/UTI settings. Multidrug-Resistant (MDR) strainswere tested (n = 12 studies, 50.0%), explicitly focused on MDR clinical isolates with particular emphasis on methicillin-resistant *S. aureus* (MRSA), extended-spectrum β-lactamase (ESBL)-producing Enterobacteriaceae, and *carbapenem*-resistant pathogens (Supplementary Table S2).

Most studies used *in vitro* antimicrobial models, specifically agar well diffusion, disc diffusion, and broth microdilution tests. Extracts comprised ethanolic, methanolic, aqueous, and hydroalcoholic preparations. Concentration ranges varied considerably between studies and were expressed as MIC and MBC values. Standard antibiotics including ciprofloxacin, gentamicin, and ampicillin were frequently utilized as positive controls, although reporting of negative controls was uneven.”

The risk of bias assessment indicated overall moderate methodological quality, with values ranging from 10 to 18. Of the 24 studies, 2 were high quality (≥17), 16 intermediate (Walia and Arora, 2013; Bag et al., 2013; Prajapati et al., 2020; Bulbul et al., 2022), and 5 low (≤12). While most studies adequately reported test systems, study design, and outcomes, common limitations included a lack of purity and physicochemical characterization of test substances, insufficient reporting of strain sources, and the absence or uncertainty of replicates and statistical analysis, which may affect reproducibility and reliability (Supplementary Table S3).

#### Antimicrobial activity of Terminalia chebula Retz

The antimicrobial efficacy of *T. chebula* extracts was evaluated against a wide range of pathogens, including *S. aureus, Klebsiella pneumoniae, Pseudomonas aeruginosa,* and *Acinetobacter baumannii*. Across the included studies, *T. chebula* demonstrated broad-spectrum antibacterial activity, with effectiveness varying based on extraction solvent and bacterial species. Overall, ethanolic and methanolic extracts exhibited superior antimicrobial activity compared to aqueous and non-polar extracts, suggesting that polar organic solvents are more efficient in extracting bioactive metabolites.

### MIC (minimum inhibitory concentration)

**FIGURE 3:**
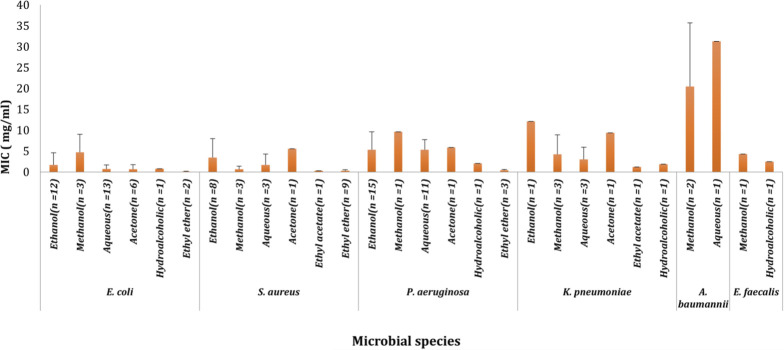
Minimum Inhibitory Concentration (MIC) of plant extract solvents against ESKAPE Pathogens.

Among the included studies, 10 studies reported minimum inhibitory concentrations (MIC) values for different solvent extracts (aqueous, ethanol, methanol, acetone, and hydroalcoholic) of *T. chebula.* The MIC values ranged from 0.03125 mg/mL to 12.5 mg/mL, indicating concentration-dependent antibacterial activity. Ethanol and methanol extracts demonstrated lower MIC values (≤5 mg/mL) against key pathogens such as *E. coli, S. aureus, and E. faecalis*) were as *P. aeruginosa* and *K. pneumoniae exhibited relatively higher MIC values (*0.67–12.5 mg/mL), reflecting inherent resistance mechanisms. *Acinetobacter baumannii* showed variable susceptibility, with some studies reporting low MIC values (0.03125 mg/mL), indicating potential sensitivity under optimized conditions. Overall, MIC data indicate that solvent polarity plays a crucial role in determining antimicrobial efficacy, with polar solvents yielding more potent extracts (Table 1; Figure 3).

**TABLE 1:** Minimum Inhibitory Concentration (MIC) values of different solvent extracts against ESKAPE Pathogens.

Solvent	Microorganisms	n	Minimum (mg/mL)	Maximum (mg/mL)	Mean (mg/mL)	Std deviation
Ethanol	*E. coli*	12	0	10	1.715	2.848
	*S. aureus*	8	0.001	12.5	3.464	4.508
	*P. aeruginosa*	15	0.109	12.5	5.320	4.310
	*K. pneumoniae*	01	0.39	12.5	12.11	-
Methanol	*E. coli*	3	1.5	9.63	4.719	4.319
	*S. aureus*	3	0.189	1.51	0.629	0.762
	*K. pneumoniae*	3	1.509	9.63	4.216	4.688
	*P. aeruginosa*	1	0	9.63	9.63	-
	*A. baumannii*	2	9.63	31.25	20.44	15.28
	*E. faecalis*	1	0	4.27	4.27	-
Aqueous	*E. coli*	13	0	2.7	0.721	0.962
	*S. aureus*	3	0.13	3.7	1.659	2.633
	*P. aeruginosa*	11	1.8	9.3	5.314	2.446
	*K. pneumoniae*	3	0.556	6.25	3.010	2.927
	*A. baumannii*	1	0	0.03125	0.03125	-
Acetone	*E. coli*	6	0	3.12	0.650	1.119
	*P. aeruginosa*	1	0.39	6.25	5.86	-
	*K. pneumoniae*	1	3.12	12.50	9.38	-
	*S. aureus*	1	0.78	6.25	5.57	-
Ethyl acetate	*S. aureus*	1	0	0.306	0.306	-
	*K. pneumoniae*	1	0	1.225	1.225	-
Hydroalcoholic	*E. coli*	1	0	0.76	0.76	-
	*P. aeruginosa*	1	0	2.08	2.08	-
	*K. pneumoniae*	1	0	1.85	1.85	-
	*E. faecalis*	1	0	2.49	2.49	-
Ethyl ether	*P. aeruginosa*	3	0.312	0.625	0.416	0.180
	*S. aureus*	9	0.078	0.625	0.322	0.206
	*E. coli*	2	0.078	0.156	0.117	0.055

### MBC (minimum bactericidal concentration)

**FIGURE 4:**
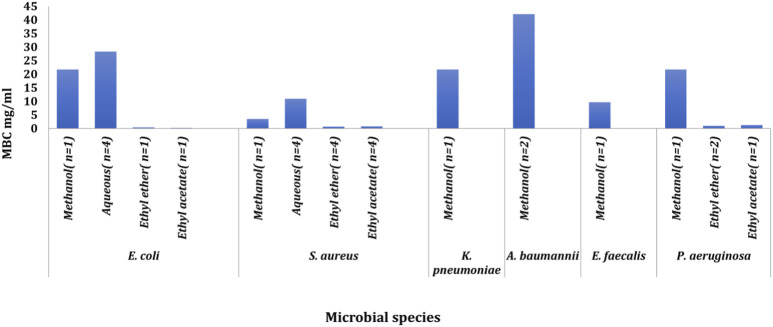
Minimum Bacterial Concentration (MBC) of plant extract solvents against ESKAPE Pathogens.

Only four studies reported MBC values, highlighting a limitation in available data. Methanolic fraction demonstrated moderate bactericidal activity (6–8 mg/mL) were as the aqueous fraction required significantly higher concentrations (12–16 mg/mL) against *E. coli* and *S. aureus*, indicating lower efficacy. Ethyl ether and ethyl acetate fractions exhibited comparatively lower MBC values (2–6 mg/mL), suggesting stronger bactericidal potential in certain fractions. The ethyl ether and ethyl acetate fractions showed modest MBCs (2–6 mg/mL) against *P. aeruginosa*, while the methanolic fraction showed higher MBC values (15 to >20 mg/mL). The methanolic fraction showed moderate bactericidal activity against *K. pneumoniae* and *E. faecalis*, with MBCs ranging from around 10–16 mg/mL. Among all solvent fractions, *A. baumannii* showed the most resistance, with MBC values of 0.0625 mg/mL. Overall, these results show that antibacterial activity is highly solvent-dependent, with *A. baumannii* remaining relatively resistant and non-polar and moderately polar fractions showing greater activity (Table 2; Figure 4).

**TABLE 2:** Minimum Bacterial Concentration (MBC) values of different solvent extracts against ESKAPE Pathogens.

Solvent	Microorganisms	n	Minimum	Maximum	Mean (mg/mL)	Std. deviation
Methanol	*E. coli*	1	0	1.51	1.51	-
	*S. aureus*	1	0	0.67	0.67	-
	*K. pneumoniae*	1	0	4.27	4.27	-
	*P. aeruginosa*	1	0	1.51	1.51	-
	*A. baumannii*	1	0	4.27	4.27	​
	*E. faecalis*	1	0	3.41	3.41	-
Aqueous	*E. coli*	4	12.5	50	28.25	15.69
	*S. aureus*	4	3.12	12.5	10.93	10.36
Ethyl ether	*E. coli*	1	0	0.312	0.312	-
	*P. aeruginosa*	2	0.625	1.25	0.937	0.441
	*S. aureus*	4	0.312	1.25	0.624	0.442
Ethyl acetate	*E. coli*	1	0	0.156	0.156	-
	*P. aeruginosa*	1	0	1.25	1.25	-
	*S. aureus*	4	0.312	1.25	0.703	0.393

A few studies reported the antimicrobial activity of isolated metabolites. Potency increases dramatically when specific bioactive molecules are isolated. The gallotannin1, 2, 6-tri-O-galloyl-β-D-glucopyranose isolated from *T. chebula* fruit demonstrated exceptional activity against multidrug-resistant (MDR) pathogens, with MIC50 values as low as 12.1 μg/ml for *S. aureus* and 15.15 μg/ml for *E. coli*. Similarly, isolated alkaloids yielded some of the lowest reported MICs at 39 μg/ml against *P. aeruginosa*, *E. coli*, and *K. pneumoniae* (Bag et al., 2012; Singh D. et al., 2012).

The relationship between MIC and MBC (the MIC Index) indicates whether an extract is bactericidal or bacteriostatic. An MBC/MIC ratio of ≤4 is generally considered bactericidal. Many *T. chebula* extracts maintain this ratio, particularly against Gram-positive *cocci* like *S. aureus* (Selma et al., 2014; Humphries et al., 2018).

### Agar well diffusion method

A total of 12 studies employed the agar well diffusion method to evaluate antibacterial activity. Hydroalcoholic and methanolic extracts produced larger zones of inhibition (22–28 mm) against *E. faecalis* and *S. aureus K. pneumoniae* showed moderate susceptibility (18–19 mm). *Pseudomonas aeruginosa* exhibited comparatively smaller zones of inhibition (15–16 mm) indicating reduced susceptibility. Overall, polar solvents consistently demonstrated greater antibacterial extraction activity (Supplementary Table S4).

### Disc diffusion method

Disc diffusion assays were performed in a smaller subset of studies (n = 2). Inhibition zones for *S. aureus* ranged from 9 to 17 mm, with ethanol and dimethyl formamide extracts having the highest mean values *K*. *pneumoniae* inhibition was rather uniform among solvents (15–18 mm), with ethanol creating the largest reported zone. *Acinetobacter baumannii* exhibits moderate to strong antibacterial activity, notably in aqueous and methanolic extracts (20–24 mm). While, crude extracts in some studies showed no activity against *Enterobacter,* isolated alkaloids demonstrated significant inhibition with a zone of 17.50 mm. These findings further support the superior efficacy of polar and semi-polar extraction systems (Supplementary Table S5).

## Discussion

The present systematic review provides a comprehensive evaluation of antimicrobial potential of *T. chebula* against clinically significant bacterial pathogens, with particularly emphasis on ESKAPE pathogens. These findings collectively demonstrated that *T. chebula* exhibits broad–spectrum antibacterial activity, with enhanced efficacy against gram-positive organisms, particularly *S. aureus*, including multidrug-resistant (MDR) strains. Plant-derived antimicrobials continue to receive widespread interest as alternative and supplementary treatment approaches to combating the rising problem of antimicrobial resistance. *Terminalia chebula* has emerged as a promising alternative because of its high metabolite content, particularly high phenolic and tannin content, which is responsible for its broad-spectrum antibacterial activity, as demonstrated in several *in vitro* studies. A structured, organism- and solvent-wise study indicates that its antibacterial activity is not uniform instead, it varies depending on the bacterial species tested and the extraction solvent utilized, with polar organic solvents like methanol and ethanol consistently outperforming the aqueous extracts, which in turn underlines the significance of methodological aspects in biological outcomes (Khan et al., 2022; Ahuja et al., 2015).

Among the organisms investigated, *S. aureus, including* the methicillin-resistant (MRSA) isolates, was consistently the most sensitive to *T. chebula* extracts. Several studies have demonstrated that hydro alcoholic, ethanolic, or methanolic extracts exhibit lower values of MIC and larger zones of inhibition for *S. aureus*. Although crude extracts show promising inhibition activity, bioactive metabolites such as gallotannins, like 1,2,6-tri-O-galloyl-β-D-glucopyranose, show remarkable inhibition activity, with MIC50 values dropping to as low as 12.1 μg/ml for MDR strains of *S. aureus*. These results show *T. chebula* extract’s remarkable ability to inhibit clinically relevant strains of *S. aureus*, thereby justifying its use as an antimicrobial agent to treat infections caused by MDR strains of *S. aureus* (Bonjar, 2004).

*Escherichia coli,* as well as *K. pneumoniae*, demonstrated a range of moderate to varying susceptibility, depending upon the strains of the organisms as well as the method of preparation of the extract. The antibacterial activity of the extract against these organisms was found to be heavily dependent upon the type of solvent used, as alcoholic as well as hydro alcoholic extracts of the plants showed lower values of MIC compared to aqueous extracts against MDR strains of *E. coli* and *Klebsiella pneumonia*. Nevertheless, consistent suppression observed with optimized solvent systems supports the antibacterial potential of *T. chebula* against these urinary tract infections and gastrointestinal infections by *E. coli* (Sharma and Kaushik, 2013).

In contrast, *P. aeruginosa* was consistently identified as the least susceptible organism, with most studies reporting significantly higher MIC and MBC values. Aqueous extracts generally exhibited minimal activity; however, hydroalcoholic and ethanolic extracts demonstrated considerable inhibitory effects, including against multidrug-resistant and carbapenem-resistant bacteria. However, the study demonstrated significant antibacterial activity against imipenem and meropenem-resistant clinical isolates, with MICs ranging from 1.6 to 12.5 mg/mL (Thirunavukkarasu et al., 2021). Furthermore, *T. chebula* extracts and isolated alkaloids function effectively as antibiotic-potentiating effect, exhibiting synergy with conventional antibiotics such as gentamicin and trimethoprim to overcome multidrug-resistance mechanisms (Bag et al., 2013). Crucially, while not initially highlighted, MBL-producing *A. baumannii* was found to be highly susceptible to fruit extracts with an MIC of 31.25 μg/ml, indicating that solvent optimization and the use of purified fractions are critical when targeting highly resistant ESKAPE pathogens (Selma et al., 2014). These findings are particularly significant given the clinical difficulty in managing *P. aeruginosa* infections.

Solvent-wise analysis reveals a consistent hierarchy of antibacterial potency, where hydroalcoholic (70% aqueous ethanol) and methanolic extracts exhibited strong activity. These solvent systems yielded larger zones of inhibition and the lowest MIC values across several bacterial species, including MDR uropathogens and ESKAPE pathogens. Ethanolic and methanolic extracts showed comparable efficacy, whereas aqueous extracts exhibited weaker and less consistent effects. Mixed-polarity solvent systems outperform other solvent systems because they can extract a wide range of bioactive metabolites, such as hydrolysable tannins (e.g., gallotannins), phenolic acids (e.g., Gallic and ellagic acid), and flavonoids, all of which are known to contribute to antimicrobial action in *T. chebula* (Khan et al., 2022).

Some studies were conducted to assess the interactions of *T. chebula* extracts with standard antibiotics like gentamicin, trimethoprim, and streptomycin. The results were promising and indicated that *T. chebula* exhibit antibiotic-potentiating and limited synergistic effects. On checkerboard titration, fruit extracts showed enhanced antibacterial activity and synergistic interaction with gentamicin and 75% synergy with trimethoprim against clinical isolates of multidrug-resistant uropathogenic *E. coli.* Moreover, the isolated gallotannin 1,2,6-tri-O-galloyl-β-D-glucopyranose displayed synergy with these antibiotics. The antibacterial activity of this metabolite may be due to its iron-chelating activity, which may remove vital minerals from bacteria (Bag et al., 2013; Bag et al., 2012). *Terminalia chebula may have potential* resistance-modifying effect as indicated by enhanced activity of antibiotics such as novobiocin against *A. baumannii* (Phatthalung et al., 2012). These findings collectively indicate that *T. chebula* may be used to augment the activity of existing antibiotics, which is of utmost importance in today’s antimicrobial resistance crisis. However, only a few studies used standardized procedures like checkerboard and FIC analysis, implying that evidence for real synergy is limited.

Although this is promising, the literature still faces issues in terms of proper methodology. Out of the studies, only 41.7% provided quantitative data, along with statistics, in the form of “mean ± SD.” The rest were based on descriptive results, with little statistical analysis. This is because, in most studies, the results differ in the form and level of data, making it difficult to conclude. In the future, it is recommended that studies follow proper experimental designs, such as those by CLSI, which include proper control groups, repetition, and statistical analysis. Such measures are required to validate and strengthen the existing evidence supporting *T. chebula* antibacterial activity (Humphries et al.). Implementing bioassay-guided fractionation and HPLC analysis is essential to isolate and validate the specific bioactive principles responsible for the plant’s therapeutic efficacy.

Beyond *T*. *chebula*, numerous plant-derived extracts have shown significant antibacterial efficacy against ESKAPE infections, including multidrug-resistant strains. A comparative study of nine medicinal plants, *Anogeissus acuminata, Punica granatum (pomegranate),* and *Soymidafebrifuga, which* were found to be even more effective than *T. chebula* leaf extracts in inhibiting a broad spectrum of MDR uropathogens, including *S. aureus* and *P. aeruginosa*. Polyphenol-rich pomegranate peel extracts demonstrated strong *in vitro* activity against *S. aureus*, including MRSA, although larger doses were required for *P. aeruginosa* and *A. baumannii* (Mishra et al., 2017; Scaglione et al., 2024). Extracts from *Leonotisocymifolia* and *Cistus* species demonstrated broad-spectrum action, with bacteriostatic or bactericidal effects determined by solvent choice and MBC/MIC ratios (Matlou et al., 2025; Zalegh et al., 2022).

A few studies explore the adjuvant potential of *T. chebula* when administered alongside conventional antibiotics. This represents a clear research gap in this regard, considering the potential for this plant to enhance the efficacy of antibiotics through synergistic interactions. Further research in this regard can lead to new strategies for tackling resistance in antibiotics, particularly when dealing with multidrug-resistant strains.

As a result, the evidence, derived from both organism as well as solvent-based extract methods, indicates *T. chebula* demonstrate broad-spectrum antimicrobial potential, which becomes even more pronounced, particularly when polar as well as mixed polarity solvents, such as methanol as well as 70% ethanol, are employed to extract bioactive tannins and phenolic metabolites. In contrast, pure water extracts as well as non-polar solvents, such as chloroform and petroleum ether, are much less active. To make this more clinically applicable, future studies are required to go beyond merely testing crude extracts, as standardized methods, such as CLSI, along with thorough toxicity profiling, are required to be performed. In this regard, some noteworthy brine shrimp lethality assays suggest that, as a result, these extracts are not only non-toxic at therapeutic doses but, as a final step, mammalian cell line assays are required to be performed, as HPLC-based phytochemicals profiling, along with synergistic antibiotic reactivation, becomes a crucial step to position *T. chebula* as a potential adjunctive measure in the global fight against antimicrobial resistance.

It is critical to evaluate the conclusions of this review with regard of specific concerns. Most of the included studies utilize *in vitro* antimicrobial assays, which are useful for preliminary screening but do not fully duplicate the complexity of *in vivo* biological systems. As a result, the pharmacological and therapeutic value of these findings is restricted unless further validated using animal models and clinical trials. Furthermore, significant variation was detected between studies in terms of extraction procedures, concentration ranges, assay settings, and reporting standards, which may have an impact on the reproducibility and comparability of results. Although most studies used conventional antibiotics as positive controls, there was some inconsistency in reporting negative controls and experimental uniformity. Furthermore, whereas some research reported the use of *T*. *chebula*, precise botanical verification was not universally recorded, indicating an additional restriction.

## Limitations and future research directions

Despite the promising results, this review has several limitations. The included research are primarily based on *in vitro* antimicrobial testing, which may not accurately reflect *in vivo* biological complexity. There is significant variety between studies in terms of extraction procedures, concentration ranges, test settings, and reporting standards, which limits direct comparability. Furthermore, irregular reporting of experimental controls and botanical verification may undermine the reliability of conclusions. To investigate active chemical metabolites, future research should use standardized experimental techniques, comprehensive phytochemical characterization, and bioassay-guided fractionation. Importantly, well-designed *in vivo* studies and clinical trials are needed to establish *T. chebula’s* therapeutic potential and safety as a resistance-modifying drug.

## Conclusion

This systematic review has identified *T*. *chebula* as a promising antibacterial agent from the realm of natural products and its extracts as having a broad spectrum of antibacterial activity against clinically relevant and multidrug-resistant bacterial infections. Organism-specific and solvent-specific analysis has shown the antibacterial potential of the extracts to be highly dependent on the solvent system and the microorganism. All the extracts were found to be more effective when they were in the hydro alcoholic, ethanolic, and methanolic solvent systems compared to the aqueous solvent system. The differences in the minimum inhibitory concentration (MIC) and the minimum bactericidal concentration (MBC) values obtained in the various studies show the complex nature of the metabolites present in the *T. chebula* extracts and the need for optimized solvent systems to achieve the maximum antibacterial potential. The antibacterial potential of the *T. chebula* extracts was found to be solvent-dependent in the ESKAPE pathogens using the disc diffusion and well diffusion methods. The results were found to be more favorable in the polar solvent systems. *Terminalia chebula* was found to show good synergism with the commonly used antibacterial agents such as gentamicin and trimethoprim. However, these findings are mostly based on *in vitro* studies and must be evaluated with care. To establish *T. chebula’s* therapeutic potential and safety as a resistance-modifying drug, more studies with standardized experimental techniques, *in vivo* validation, and well-designed clinical investigations are required.”
