# Supplementary material for: Terminalia chebula Retz. As a resistance-modifying botanical drug against priority pathogens: a systematic review
Source: Front Pharmacol. 2026 Jun 9;17:1855899. doi: 10.3389/fphar.2026.1855899 (PMC13288416; doi:10.3389/fphar.2026.1855899)
Supplement: Supplementary file 1 [file Supplementaryfile1.docx]

**Supplementary file**

Supplementary material containing search keywords, summary tables of included studies, and results of well diffusion and disc diffusion assays of solvent-based extracts against ESKAPE pathogens.

**Supplementary table 1. Database-wise search syntax, keywords, and Boolean operators used for systematic literature retrieval.**

**Keywords:**

| **Sl. No.** |  | **Keywords** |
| --- | --- | --- |
| **1** | **Organisms** | *E. coli, Escherichia coli, P. aeruginosa, Pseudomonas aeruginosa, K. pneumoniae, Klebsiella pneumoniae, A. baumannii, Acinetobacter baumannii*, ESKAPE. |
| **2** | ***Terminalia chebula*** | *Terminalia chebula Retz*, T. chebula, Haritaki, Terminalia chebula fruit extract, Black myrobalan, Harara, Harada, fructus chebulae, chebulae fructus, Terminalia chebula fruit, chebulae extract, Terminalia chebula extract, Black myrobalan, Chebulic myrobalan, Harad, Harara, Harra, Abhaya, Girija, Pathya, Rohini, Siva, Sudha, Habra, Hirada, Divya, Kayastha, Kadu, Nechi, Seya, Siva. |
| **3** | **Antimicrobial** | Antimicrobial activity, Antimicrobial Potential, Antibacterial activity, Antibacterial potential, antimicrobial resistance, AMR, Antibiotic resistance, Multidrug Resistance, MDR, Pathogen resistance, microbial resistance, Resistance to Antibiotics, Pharmaco resistance, Bacterial Priority pathogens. |

**Search words for:**

| **PUBMED(https://pubmed.ncbi.nlm.nih.gov/)** |
| --- |
| *(("E. coli" OR "Escherichia coli" OR "P. aeruginosa" OR "Pseudomonas aeruginosa" OR "K. pneumoniae" OR "Klebsiella pneumoniae" OR "A. baumannii" OR "Acinetobacter baumannii" OR "Priority pathogens" OR ESKAPE)* **AND** ((chebulae fruit [MeSH Terms]) OR "Terminalia chebula Retz" OR "T. chebula" OR Haritaki OR "Terminalia chebula fruit extract" OR "Black myrobalan" OR Harara OR Harada OR "fructus chebulae" OR "chebulae fructus" OR "Terminalia chebula fruit" OR "chebulae extract" OR "Terminalia chebula extract" OR "Black myrobalan" OR "Chebulic myrobalan" OR Harad OR Harara OR Harra OR Abhaya OR Girija OR Pathya OR Rohini OR Siva OR Sudha OR Harra OR Habra OR Hirada OR Divya OR Kayastha OR Kadu OR Nechi OR Seya OR Siva)) **AND** ("Antimicrobial activity" OR "Antimicrobial Potential" OR "Antibacterial activity" OR "Antibacterial potential" OR "Antimicrobial resistance" OR AMR OR "Antibiotic resistance" OR "Multidrug Resistance" OR MDR OR "Pathogen resistance" OR "microbial resistance" OR "Resistance to Antibiotics“ OR “Pharmaco resistance” OR “Bacterial Priority pathogens”) **- 108 hits** |
|  |
| **SCOPUS(https://www.scopus.com/)** |
| (TITLE-ABS-KEY ("E. coli" OR "*Escherichia. coli*" OR "P. aeruginosa" OR "*Pseudomonas. aeruginosa"* OR "*K. pneumoniae*" OR "*Klebsiella. pneumoniae*" OR "*A. baumannii*" OR "*Acinetobacter. baumannii"* OR ESKAPE)) **AND**(TITLE-ABS-KEY ("Terminalia chebula Retz" OR "T. chebula" OR Haritaki OR "Terminalia chebula fruit extract" OR "Black myrobalan" OR Harara OR Harada OR "fructus chebulae" OR "chebulae fructus" OR "Terminalia chebula fruit" OR "chebulae extract" OR "Terminalia chebula extract" OR "Black myrobalan" OR "Chebulic myrobalan" OR Harad OR Harara OR Harra OR Abhaya OR Girija OR Pathya OR Rohini OR Siva OR Sudha OR Harra OR Habra OR Hirada OR Divya OR Kayastha OR Kadu OR Nechi OR Seya OR Siva)) **AND** (TITLE-ABS-KEY ("Antimicrobial activity" OR "Antimicrobial Potential" OR "Antibacterial activity" OR "Antibacterial potential" OR "Antimicrobial resistance" OR AMR OR "Antibiotic resistance" OR "Multidrug Resistance" OR MDR OR "Pathogen resistance" OR "microbial resistance" OR "Resistance to Antibiotics")) – **123 hits** |
|  |
| **SCIENCE DIRECT(https://www.sciencedirect.com/)** |
| ("*Escherichia coli" OR "Pseudomonas aeruginosa" OR "Klebsiella pneumoniae" OR "Acinetobacter baumannii"* OR ''ESKAPE'') **AND** ("Terminalia chebula'')**AND** (“antimicrobial activity” OR “antibiotic resistance” OR” Multidrug resistance”) –**434 hits** |
|  |
| **GOOGLE SCHOLAR(https://scholar.google.com/)** |
| (("*E. coli" OR "Escherichia coli" OR "P. aeruginosa" OR "Pseudomonas aeruginosa" OR "K. pneumoniae" OR "Klebsiella pneumoniae" OR "A. baumannii" OR "Acinetobacter baumannii" OR "Priority pathogens" OR ESKAPE*) **AND** ("Terminalia chebula Retz" OR "T. chebula" OR Haritaki OR "Terminalia chebula fruit extract" OR "Black myrobalan" OR Harara OR Harada OR "fructus chebulae" OR "chebulae fructus" OR "Terminalia chebula fruit" OR "chebulae extract" OR "Terminalia chebula extract" OR "Black myrobalan" OR "Chebulic myrobalan" OR Harad OR Harara OR Harra OR Abhaya OR Girija OR Pathya OR Rohini OR Siva OR Sudha OR Harra OR Habra OR Hirada OR Divya OR Kayastha OR Kadu OR Nechi OR Seya OR Siva) **AND** ("Antimicrobial activity" OR "Antimicrobial Potential" OR "Antibacterial activity" OR "Antibacterial potential" OR "Antimicrobial resistance" OR AMR OR "Antibiotic resistance" OR "Multidrug Resistance" OR MDR OR "Pathogen resistance" OR "microbial resistance" OR "Resistance to Antibiotics“ OR “Pharmaco resistance” OR “Bacterial Priority pathogens”)**– 3,430 hits** |

| **Sr.no** | **Study ID** | **Plant part used** | **Collection place (location)** | **Extraction method** | **Solvent used** | **Organism used** | **Methods** | **No. of replicate** | **Antibiotic used** | **Outcome of the study** |
| --- | --- | --- | --- | --- | --- | --- | --- | --- | --- | --- |
| 1 | **Bag *et al.,* (2009)** | Fruit | Kolkata | Maceration | Ethanol, cold and hot aqueous | *S.aureus*  *E. coli* and clinical samples  *MRSA, SXT/ TMP-resistant UPEC* | MIC, MBC, Well diffusion | 3 | Gentamicin | The ethanolic extract was most effective against resistant *E.coli*(zone: 21 mm; MIC: 3.12 mg/ml), while the aqueous extract (Boiled) showed the highest activity against MRSA (zone: 19–20 mm; MIC: 3.12mg/ml). This study has not deciphered the mean and SD values. |
| 2 | **Ahuja *et al.,* (2014)** | Fruit | Patiala, Punjab | Soxhlet | Ethanol,  Chloroform,  Acetone | *S.aureus*  *E.coli* | Well diffusion | - | Amoxicillin | *Escherichia coli* and *Staphylococcus aureus* inhibition was observed at 100% concentration and produced inhibition zones of 24mm and 22 mm, respectively. This study does not mention the mean and SD values. |
| 3 | **Gupta *et al.,* (2023)** | Fruit | Punjab | Soxhlet | Methanol, ethanol,  Acetone, water | *S.aureus(MTCC 7443),*  *E. coli* | Well diffusion | 3 | - | Themethanolic extracts exhibited the strongest antibacterial activity, with a maximum inhibition zone of 25.6 ± 0.57mm against *E.coli* at 100% concentration. This study has given the mean and SD values. |
| 4 | **Jinukuti*et al.,* (2013)** | Fruit | Hyderabad | Maceration | Aqueous | *S.aureusMTCC (7443),*  *E.coli MTCC (7410),*  *P.aeruginosaMTCC (2295),*  *K.pneumoniaeMTCC (3384)* | Well Diffusion | 3 | Amikacin | The aqueous extract showed antimicrobial activity, notably against gram-negative ESKAPE pathogens. The Mean and SD valuesare revealed for this study. |
| 5 | **Mostafa*et al.* (2011)** | Leaf | Dhaka | Maceration | Methanol  Aqueous  Ethanol  Ethyl acetate  Chloroform | *E. coli* | MIC/MBC/  Disc diffusion | - | Ampicillin, streptomycin  Chloramphenicol,  Ciprofloxacin  Nalidixic acid,  Trimethoprim,  Rifampicin, polymyxin B, ceftriaxone, oxytetracycline | Methanolic and aqueous extracts inhibited *E. coli with* MIC and MBC values between 6–8 mg/ml and SD values were not revealed in the study. |
| 6 | **Prasuna *et al.,* (2016)** | Fruit | Telangana | Maceration | Ethanol  Ethyl acetate  Acetone  Methanol | *E. coli*  *P. aeruginosa*  *S.aureus* | Disc diffusion, Broth Micro-dilution | - | Streptomycin | Methanolic extract showed the highest activity against *E.coli*(14 mm inhibition zone; MIC: 1 mg/ml). The study reports the mean and SD for each concentration. |
| 7 | **Naqvi *et al.,* (2010)** | Dried whole plant | Hyderabad,  Pakistan | Maceration | Ethanol  Aqueous  Chloroform  Ethyl acetate  n-butanol | *E. coli*  *S. aureus*  *K. pneumoniae*  *P. aeruginosa* | Well diffusion (crude+  fractions) | 3 | Amoxicillin | Crude extract showed moderate to significant antibacterial activity against *S.aureus*and*P.aeruginosa*but was ineffective against *E.coli*and *K. pneumoniae*. Among solvent fractions, aqueous, ethyl acetate, and n-butanol showed activity primarily against Gram-positive pathogens, while Gram-negative ESKAPE bacteria were mostly resistant. The Mean and SD values for this study are not revealed. |
| 8 | **Bag *et al.,* (2013)** | Fruit | Kolkata | Maceration | Hydroalcoholic | *E.coli (ATCC- 8739),*  *9- E. coli,*  *6- P. aeruginosa,*  *8- K. pneumonia,*  *5- E. faecalis* | Well diffusion, Micro broth dilution | 3 | Multi drugs | Hydroalcoholic extracts demonstrated the strongest antibacterial activity against ESKAPE pathogens, with inhibition zones up to 23.62 ± 1.24 mm and MIC₅₀ values as low as 0.24 mg/ml against *E. coli*. The Mean and SD values for well diffusion analysisare mentioned in the study. |
| 9 | **Parekh *et al.,* (2008)** | Fruit | Rajkot | Maceration | Petroleum ether  Chloroform  Dimethylformamide  Ethanol  Water | *K.pneumoniae(NCIM 2719),*  *S.aureus(ATCC 25923),*  *S.aureus(ATCC 29737),*  *E.coli (ATCC 25922),*  *P.aeruginosa (ATCC 27853)*  *Enterobacter aerogenes* | Disc diffusion | 3 | Piperacillin, Gentamicin, and Amphotericin B | The ethanol extract exhibited the maximum antibacterial activity against ESKAPE pathogens, while the petroleum ether and chloroform extracts showed minimal activity. Mean and SD values are not given in the study. |
| 10 | **Tariq *et al.,* (2012)** | Fruit | Bengali, Karnataka | Soxhlet | Acetone  Ethanol  Cold and  Hot aqueous | *E.coli 1-5* | Disc diffusion, microdilution | - | Multi drugs | Acetone and ethanolic extracts exhibited superior antibacterial activity against multidrug-resistant *E.coli*Uropathogenic strains, with MICs as low as 0.488–0.977 mg/ml.  Mean and SD values are not given in the study. |
| 11 | **Salma H*et al.,* (2014)** | Fruit | Algeria | Maceration  Decoction | Methanol  Aqueous | *A. baumannii* | Disc diffusion, MIC, MBC | 3 | Gentamicin | Both methanolic and aqueous extracts inhibited metallo-beta-lactamases (MBLs) produced by *Acinetobacter baumannii*, with IC₅₀ values of 0.0184 mg/ml and 0.0196mg/ml, respectively. Identical MIC (0.03125mg/ml) and MBC (0.0625mg/ml) values were observed. |
| 12 | **Bag *et al.,* (2010)** | Fruit | Kolkata | Maceration | Ethanol | *E.coli (ATCC 8739),*  *8 MDR E. coli* | Microdilution, checkerboard, time-kill assay | - | Amoxicillin, Ciprofloxacin, Gentamicin, Ceftazidime, and Trimethoprim | The ethanolic extracts demonstrated synergistic antibacterial effects with gentamicin (87% of isolates) and trimethoprim (75%) against multidrug-resistant *E.coli*uropathogenic strains, and additive effects with ciprofloxacin, amoxicillin, and ceftazidime. Mean and SD values are not given in the study. |
| 13 | **Bag *et al.,* (2012)** | Fruit | Kolkata | Maceration | Acetone  Ethanol  Aqueous | *E.coli (ATCC 8739),*  *S.aureus(ATCC6538),*  *21- E. coli,*  *9- P. aeruginosa,*  *16- K. pneumonia,*  *6- S. aureus* | Well diffusion, microdilution, and kill kinetics | 3 | Ciprofloxacin, Gentamicin | The ethanolextract exhibited the highest antibacterial activity against multidrug-resistant uropathogenic bacterial isolates, with inhibition zones up to 24.51 ± 1.28 mm and MIC₅₀ values ranging from 0.20–0.975 mg/ml. Kill-kinetics revealed dose- and time-dependent bactericidal activity against *E.coli*, with >3 log₁₀ CFU/ml reduction at 4×MIC within 24h. The mean and SD values for well diffusion analysis is given in this study. |
| 14 | **Anand S*et al.,* (2012)** | Fruit | Shimla | Maceration | Aqueous | *E. coli*  *S. aureus* | Well diffusion | 3 | - | The aqueous extract demonstrated the highest antibacterial activity against *S.aureus*(17.77 mm) and *E.coli*(16.66 mm). The mean and SD values of respective concentrations are mentioned. |
| 15 | **Phatthalung *et al.* (2012)** | Fruit | Thailand | Maceration | Ethanol | *A. baumannii (ATCC 19606)* | Microdilution, percentage growth inhibition | - | - | The ethanolic extract, along with other extracts, significantly enhanced the activity of novobiocin against *Acinetobacter baumannii* at 250µg/ml and 1 µg/ml novobiocin. The mean and SD values have not been given for the study. |
| 16 | **Singh *et al.,* (2012)** | Leaf  Stem  Bark  Fruit | Bangalore | Soxhlet | Hot methanol, petroleum ether, ethyl ether, ethyl acetate | *E.coli (MTCC 46)*  *P.aeruginosa(MTCC 1934)*  *S.aureus(MTCC 3160)*  *E.aerogenes* | Disc diffusion, microdilution, MBC | 3 | Streptomycin | The free flavonoid extract exhibited the highest antimicrobial activity, with a max zone of inhibition of 20.75 mm against *P. aeruginosa*. The MIC of the extracts ranged from 0.039 to 0.625 mg/ml, and the MBC/MFC ranged from 0.039 to 1.25mg/ml, demonstrating that the strongest antimicrobial potential was for theleaf alkaloids from the leaf. |
| 17 | **Tiwana *et al.,* (2024)** | Fruit | Australia | Maceration | Aqueous  Methanol  Ethyl acetate | *E.coli (ATCC 25922)*  *S.aureus(ATCC 25923)*  *MRSA (ATCC 43300)*  *K.pneumoniae(ATCC 13883)*  *ESBLK.pneumoniae(ATCC 700603)*  *ESBL E. coli, MRSA* | Disc diffusion, Microdilution,FIC | 3 | Multi-drugs | Aqueous and methanolic extracts showed substantial antibacterial activity against *S.aureus*and methicillin-resistant *S.aureus*(MRSA), with MICs ranging from 94µg/ml to 392µg/ml.Italso inhibited the growth of *K. pneumoniae* and *E. coli*, with MIC values of 556µg/ml and 755µg/ml, respectively. The mean and SD values have not been given for the study. |
| 18 | **Singh *et al.,* (2012)** | Fruit | Mumbai | Soxhlet | Ethyl acetate  Acetone  Methanol  Water | *S.aureus(MTCC)*  *P.aeruginosa(MTCC)* | MIC, MBC, Micro dilution | 3 | Gentamicin | The methanolic extract exhibited inhibition zones of 19.33 mm at 0.5 mg/100µL and 21.00 mm at 1 mg/100µL. MIC and MBC values varied among the extracts, with methanolic and aqueous showing similar results. The mean and SD values have not been given for the study. |
| 19 | **Thirunavukkarasu *et al.,* (2021)** | Fruit | Chennai | Maceration | Ethanol, hydroalcoholic | *Imipenem resistance P.aeruginosa(n=21)*  *Meropenem-resistant P. aeruginosa (n=17)* | Agar dilution method, MIC | - | Imipenem, Meropenem | The MICs ranged from 1.6 to 12.5 mg/ml for *T.chebula* against both imipenem- and meropenem-resistant *P.aeruginosa*strains. The mean and SD values have not been given for the study. |
| 20 | **Parveen *et al.,* (2018)** | Fruit | New Delhi | Maceration | Aqueous  Methanol | *S.aureus(MTCC 902)*  *E.coli (MTCC 443)*  *P.aeruginosa(MTCC 2453)* | Well diffusion, macrodilution (% MGl) | 3 | Ampicillin | Methanolic extracts showed the highest efficacy. The inhibition zones indicated significant activity against multidrug-resistant strains such as *P.aeruginosa* (4mm), *E.coli* (9mm) and *S.aureus*(25mm). |
| 21 | **Khan *et al.,* (2021)** | Fruit | Pakistan | Maceration | Ethanol | *E.coli*  *Staphylococcus sp* | Disc diffusion, MIC | 3 | Ciprofloxacin | The ethanolic was effective against *E.coli* and *Staphylococcus* species, with MIC values of 10.0 mg/ml and 2.5 mg/ml, respectively. The mean and SD values have not been given for the study. |
| 22 | **Mishra *et al.* (2015)** | Leaf/ fruits | Odisha |  | Methanol | *E. faecalis*  *S. aureus*  *A. baumannii*  *E. coli*  *K. pneumoniae*  *P. aeruginosa* | Disc diffusion, MIC, MBC | 3 | Gentamicin | Methanolic extracts exhibited the highest MIC (9.63mg/ml) and MBC (21.67mg/ml) values for most other MDR bacteria, including *E. coli*. This study has not deciphered the mean and SD values. |
| 23 | **Kathirvel A *et al.,* (2012)** | Leaf | Tamil nadu | Soxhlet | Petroleum ether  Chloroform  Ethyl acetate  Acetone  Methanol  Water | *E. faecalis*  *S. aureus*  *K. pneumoniae* | Well diffusion | 3 | Streptomycin | The acetone and ethyl acetate extracts showed strong scavenging activity, with acetone achieving an IC50 value of 0.13mg (130 µg), comparable to the standards α-tocopherol 0.197mg (197µg) and ascorbic acid 0.18 mg (180 µg). The acetone extract exhibited the most potent antibacterial activity. The study includes the respective mean and SD values. |
| 24 | **Bonjar**  ***et al.,* (2003)** | Seeds | Iran |  | Methanol | *E.coli (PTCC 1330)*  *P.aeruginosa(PTCC 1074)*  *K.pneumoniae(PTCC 1053)*  *S.aureus(PTCC 1112)* | Agar well diffusion | - | - | Extracts exhibited an MIC of 0.62 mg/ml against *Staphylococcus aureus*. *Klebsiella pneumonia* wasone of the most susceptible Gram-negative bacteria, while *Staphylococcus aureus* was the most susceptible Gram-positive bacterium. The mean and SD values have not been given for the study. |

***Supplementary Table 2: Characteristics of Included Studies. This table summarizes the key methodological details of the studies included in the systematic review, highlighting the diversity in plant material processing and target pathogen***

| **Reliability assessment of in vitro toxicity studies** | |
| --- | --- |
| **Authors:**  Kamran Zaman^†*^, Surthi Ravedar^†^, Nidhi R^†^, Kalesh Karun, Jainabbi Patel, Flemin Felix, Kranthi Kiran Akula, Nidhi Hiremath, Shivani Tendulkar, Tejaswini Salunkhe, Asif Kavathekar, Jyothi Bhat* | |
| **Title:** | ***Terminalia chebula* Retz. as a Antibiotic- potentiating Phytotherapeutic Against ESKAPE Pathogens: A Systematic Review** |

| **Authors** | **Criteria Group I: Test substance identification** | | | | **Criteria Group II: Test system characterisation** | | | **Criteria Group III: Study design description** | | | | |  | **Criteria Group IV: Study results documentation** | | | **Criteria Group V: Plausibility of study design and data** | | **Score** |
| --- | --- | --- | --- | --- | --- | --- | --- | --- | --- | --- | --- | --- | --- | --- | --- | --- | --- | --- | --- |
|  | **Was the test substance identified?** | **Is the purity of the substance given?** | **Is information on the source/origin of the substance given?** | **Is all information on the nature and/or physico-chemical properties of the test item given, which you deem indispensable for judging the data (see explanation for examples)?** | **Is the test system described?** | **Is information given on the source/origin of the test system?** | **Are necessary information on test system properties, and on conditions of cultivation and maintenance given?** | **Is the method of administration given (see explanations for details)?** | **Are doses administered or concentrations in application media given?** | **Are frequency and duration of exposure as well as time-points of observations explained?** | **Were negative controls included (give also point, if not necessary, see explanations)?** | **Were positive controls included (give also point, if not necessary, see explanations)?** | **Is the number of replicates (or complete repetitions of experiment) given?** | **Are the study endpoint(s) and their method(s) of determination clearly described?** | **Is the description of the study results for all endpoints investigated transparent and complete?** | **Are the statistical methods for data analysis given and applied in a transparent manner (give also point, if not necessary/applicable, see explanations)?** | **Is the study design chosen appropriate for obtaining the substance-specific data aimed at (see explanations for details)?** | **Are the quantitative study results reliable (see explanations for arguments)?** |  |
| Bag et al., (2009)(28) | 1 | 1 | 0 | 1 | 1 | 1 | 1 | 1 | 1 | 1 | 1 | 1 | 1 | 1 | 0 | 1 | 0 | 1 | 15 |
| Ahuja et al., (2014)(29) | 1 | 1 | 0 | 1 | 1 | 1 | 1 | 1 | 1 | 0 | 1 | 0 | 1 | 1 | 1 | 1 | 0 | 1 | 14 |
| Gupta et al., (2023)(30) | 1 | 0 | 1 | 1 | 1 | 1 | 1 | 1 | 1 | 1 | 1 | 0 | 1 | 1 | 1 | 1 | 1 | 1 | 16 |
| Jinukutiet al., (2013) | 1 | 1 | 0 | 1 | 1 | 1 | 1 | 1 | 1 | 0 | 1 | 1 | 1 | 1 | 1 | 1 | 0 | 1 | 15 |
| Mostafa et al. (2011)(32) | 1 | 0 | 1 | 0 | 1 | 0 | 1 | 1 | 1 | 1 | 1 | 1 | 0 | 1 | 1 | 0 | 1 | 0 | 12 |
| Prasuna et al., (2016) | 1 | 0 | 1 | 0 | 1 | 0 | 1 | 1 | 1 | 1 | 1 | 1 | 0 | 1 | 1 | 1 | 1 | 0 | 13 |
| Naqvi et al., (2010) | 1 | 0 | 1 | 0 | 1 | 1 | 1 | 1 | 1 | 1 | 1 | 1 | 0 | 1 | 1 | 1 | 1 | 0 | 12 |
| Bag et al., (2013) | 1 | 1 | 1 | 1 | 1 | 1 | 1 | 1 | 1 | 1 | 1 | 1 | 1 | 1 | 1 | 1 | 1 | 1 | 18 |
| Parekh et al., (2008) | 1 | 0 | 1 | 0 | 1 | 1 | 1 | 1 | 1 | 1 | 1 | 1 | 1 | 1 | 1 | 0 | 1 | 1 | 15 |
| Tariq et al., (2012) | 1 | 0 | 1 | 0 | 1 | 1 | 1 | 1 | 1 | 1 | 1 | 0 | 0 | 1 | 1 | 0 | 1 | 0 | 12 |
| Salma H et al., (2014)(38) | 1 | 0 | 1 | 1 | 1 | 1 | 1 | 1 | 1 | 1 | 1 | 1 | 1 | 1 | 1 | 1 | 1 | 1 | 17 |
| Bag et al., (2010)(39) | 1 | 0 | 1 | 0 | 1 | 1 | 1 | 1 | 1 | 1 | 1 | 1 | 1 | 1 | 1 | 1 | 1 | 1 | 16 |
| Bag et al., (2012)(25) | 1 | 0 | 1 | 0 | 1 | 1 | 1 | 1 | 1 | 1 | 1 | 1 | 1 | 1 | 1 | 1 | 1 | 1 | 16 |
| Anand S et al., (2012) | 1 | 0 | 1 | 0 | 1 | 1 | 1 | 1 | 1 | 1 | 0 | 0 | 1 | 1 | 1 | 0 | 1 | 0 | 12 |
| Phatthalung et al. (2012) | 1 | 0 | 1 | 1 | 1 | 1 | 1 | 1 | 1 | 1 | 1 | 1 | 1 | 1 | 1 | 1 | 1 | 1 | 17 |
| Singh et al., (2012) | 1 | 0 | 1 | 0 | 1 | 1 | 1 | 1 | 1 | 1 | 1 | 1 | 1 | 1 | 1 | 0 | 1 | 1 | 15 |
| Tiwana et al., (2024) | 1 | 1 | 1 | 1 | 1 | 1 | 1 | 1 | 1 | 1 | 1 | 1 | 1 | 1 | 1 | 1 | 1 | 1 | 18 |
| Singh et al., (2012) | 1 | 0 | 1 | 0 | 1 | 1 | 1 | 1 | 1 | 1 | 1 | 1 | 1 | 1 | 1 | 0 | 1 | 1 | 16 |
| Thirunavukkarasu et al., (2021) | 1 | 0 | 1 | 1 | 1 | 1 | 1 | 1 | 1 | 1 | 1 | 1 | 0 | 1 | 1 | 0 | 1 | 1 | 15 |
| Parveen et al., (2018) | 1 | 1 | 0 | 1 | 1 | 1 | 1 | 1 | 1 | 0 | 1 | 1 | 1 | 1 | 1 | 1 | 0 | 1 | 15 |
| Khan et al., (2021) | 1 | 1 | 0 | 1 | 1 | 1 | 1 | 1 | 1 | 1 | 1 | 0 | 1 | 1 | 1 | 1 | 1 | 1 | 15 |
| Mishra et al. (2015) | 1 | 1 | 0 | 1 | 1 | 1 | 1 | 1 | 1 | 0 | 1 | 0 | 1 | 1 | 0 | 1 | 0 | 1 | 13 |
| Kathirvel A et al., (2012) | 1 | 0 | 1 | 0 | 1 | 1 | 1 | 1 | 1 | 1 | 1 | 1 | 1 | 1 | 1 | 1 | 1 | 1 | 16 |
| Bonjar et al.,(2003) | 1 | 0 | 1 | 0 | 1 | 1 | 0 | 1 | 1 | 0 | 0 | 0 | 0 | 1 | 1 | 0 | 1 | 1 | 10 |

***Supplementary Table 3. Risk of bias of included in vitro studies assessed using the ToxR tool. Studies were evaluated across five domains and scored as 1 (adequate) or 0 (inadequate), with a maximum score of 18 used to classify reliability.***

**Well diffusion**

| **Organism** | **Solvents** | **n** | **Minimum(mm)** | **Maximum(mm)** | **Mean(mm)** | **Std. Deviation** |
| --- | --- | --- | --- | --- | --- | --- |
| *E. coli* | Aqueous | 19 | 3.33 | 19.88 | 12.05 | 4.39 |
|  | Acetone | 10 | 10 | 21 | 16.59 | 3.49 |
|  | Ethanol | 14 | 0 | 28.33 | 16.17 | 7.46 |
|  | Methanol | 4 | 19.3 | 25.6 | 22.3 | 2.74 |
|  | Chloroform | 4 | 5 | 15 | 9.25 | 4.34 |
|  | Hydroalcoholic | 2 | 23.62 | 26.33 | 24.97 | 1.91 |
|  | Overall | 53 | 61.25 | 136.14 | 101.35 | 24.37 |
|  |  |  |  |  |  |  |
| *S. aureus* | Aqueous (Cold & hot) | 17 | 4 | 25 | 12.88 | 5.25 |
|  | Acetone | 11 | 4 | 21 | 13.99 | 4.70 |
|  | Ethanol | 14 | 6 | 22.66 | 15.07 | 4.52 |
|  | Ethyl acetate | 1 | 10.67 | 10.67 | 10.67 | . |
|  | Methanol | 7 | 12.33 | 24 | 19.31 | 3.82 |
|  | Chloroform | 5 | 4 | 12 | 8.46 | 3.09 |
|  | Petroleum ether | 1 | 9.67 | 9.67 | 9.67 | * |
|  | Hydroalcoholic | 1 | 27.66 | 27.66 | 27.66 | * |
|  | Overall | 60 | 78.33 | 152.66 | 117.74 | 21.41 |
|  |  |  |  |  |  |  |
| *K. pneumoniae* | Aqueous | 6 | 9.27 | 23.6 | 18.14 | 5.79 |
|  | Acetone | 2 | 12.77 | 14.33 | 13.55 | 1.10 |
|  | Ethanol | 2 | 0 | 17.5 | 8.75 | 12.37 |
|  | Ethyl acetate | 1 | 13.33 | 13.33 | 13.33 | * |
|  | Methanol | 2 | 10 | 13.3 | 11.65 | 2.33 |
|  | Chloroform | 1 | 12.33 | 12.33 | 12.33 | * |
|  | Petroleum ether | 1 | 9.67 | 9.67 | 9.67 | * |
|  | Hydroalcoholic | 1 | 18.84 | 18.84 | 18.84 | * |
|  | Overall | 16 | 86.21 | 122.9 | 106.26 | 21.60 |
|  |  |  |  |  |  |  |
| *P. aeruginosa* | Aqueous | 5 | 8.85 | 19.5 | 15.46 | 4.05 |
|  | Acetone | 1 | 8.67 | 8.67 | 8.67 | * |
|  | Ethanol | 2 | 10 | 10.67 | 10.33 | 0.47 |
|  | Methanol | 3 | 4 | 16 | 11.33 | 6.42 |
|  | Hydroalcoholic | 1 | 15.46 | 15.46 | 15.46 | * |
|  | Overall | 12 | 46.98 | 70.3 | 61.25 | 10.95 |
|  |  |  |  |  |  |  |
| *E. faecalis* | Aqueous | 1 | 11.67 | 11.67 | 11.67 | * |
|  | Acetone | 1 | 20 | 20 | 20 | * |
|  | Ethyl acetate | 1 | 18.67 | 18.67 | 18.67 | * |
|  | Methanol | 1 | 19.67 | 19.67 | 19.67 | * |
|  | Chloroform | 1 | 10.33 | 10.33 | 10.33 | * |
|  | Petroleum ether | 1 | 8.33 | 8.33 | 8.33 | * |
|  | Hydroalcoholic | 1 | 22.34 | 22.34 | 22.34 | * |
|  | Overall | 7 | 111.01 | 111.01 | 111.01 | 0 |

***Supplementary Table 4. Well diffusion of solvent-based extracts against ESKAPE Pathogens***

***Note: **** *Values represent single observations; therefore, standard deviation (SD) was not calculated.*

**Disc diffusion**

| **Organism** | **Solvents** | **n** | **Minimum(mm)** | **Maximum(mm)** | **Mean(mm)** | **Std. Deviation** |
| --- | --- | --- | --- | --- | --- | --- |
| *S. aureus* | Aqueous | 2 | 14 | 15 | 14.5 | 0.70 |
|  | Dimethyl formamide | 2 | 15 | 15 | 15 | 0 |
|  | Ethanol | 2 | 15 | 17 | 16 | 1.41 |
|  | Chloroform | 2 | 11 | 12 | 11.5 | 0.70 |
|  | Petroleum ether | 2 | 9 | 9 | 9 | 0 |
|  | Overall | 10 | 64 | 68 | 66 | 2.82 |
|  |  |  |  |  |  |  |
| *K. pneumoniae* | Aqueous | 1 | 15 | 15 | 15 | * |
|  | Dimethyl formamide | 1 | 16 | 16 | 16 | * |
|  | Ethanol | 1 | 18 | 18 | 18 | * |
|  | Chloroform | 1 | 15 | 15 | 15 | * |
|  | Overall | 4 | 64 | 64 | 64 | 0 |
|  |  |  |  |  |  |  |
| *Enterobacter spp.* | Ethanol | 1 | 10 | 10 | 10 | * |
|  | Chloroform | 1 | 14 | 14 | 14 | * |
|  | Overall | 2 | 24 | 24 | 24 | 0 |

***Supplementary Table 5. Disc diffusion of solvent-based extracts against ESKAPE Pathogens***

***Note: **** *Values represent single observations; therefore, standard deviation (SD) was not calculated*

| **Sr.no** | **Study ID** | **Plant part used** | **Collection place (location)** | **Extraction method** | **Solvent used** | **Organism used** | **Methods** | **No. of replicate** | **Antibiotic used** | **Outcome of the study** |
| --- | --- | --- | --- | --- | --- | --- | --- | --- | --- | --- |
| 1 | **Salma H*et al.,* (2014)**^(38)^ | Fruit | Algeria | Maceration  Decoction | Methanol  Aqueous | *A. baumannii* | Disc diffusion, MIC, MBC | 3 | Gentamicin | Both methanolic and aqueous extracts inhibited metallo-beta-lactamases (MBLs) produced by *Acinetobacter baumannii*, with IC₅₀ values of 0.0184 mg/ml and 0.0196mg/ml, respectively. Identical MIC (0.03125mg/ml) and MBC (0.0625mg/ml) values were observed. |
| 2 | **Bag *et al.,* (2010)^(39)^** | Fruit | Kolkata | Maceration | Ethanol | *E.coli (ATCC 8739),*  *8 MDR E. coli* | Microdilution, checkerboard, time-kill assay | - | Amoxicillin, Ciprofloxacin, Gentamicin, Ceftazidime, and Trimethoprim | The ethanolic extracts demonstrated synergistic antibacterial effects with gentamicin (87% of isolates) and trimethoprim (75%) against multidrug-resistant *E.coli*uropathogenic strains, and additive effects with ciprofloxacin, amoxicillin, and ceftazidime. Mean and SD values are not given in the study. |
| 3 | **Bag *et al.,* (2012)**^(25)^ | Fruit | Kolkata | Maceration | Acetone  Ethanol  Aqueous | *E.coli (ATCC 8739),*  *S.aureus(ATCC6538),*  *21- E. coli,*  *9- P. aeruginosa,*  *16- K. pneumonia,*  *6- S. aureus* | Well diffusion, microdilution, and kill kinetics | 3 | Ciprofloxacin, Gentamicin | The ethanolextract exhibited the highest antibacterial activity against multidrug-resistant uropathogenic bacterial isolates, with inhibition zones up to 24.51 ± 1.28 mm and MIC₅₀ values ranging from 0.20–0.975 mg/ml. Kill-kinetics revealed dose- and time-dependent bactericidal activity against *E.coli*, with >3 log₁₀ CFU/ml reduction at 4×MIC within 24h. The mean and SD values for well diffusion analysis is given in this study. |
| 4 | **Tiwana *et al.,* (2024)**  ^(26)^ | Fruit | Australia | Maceration | Aqueous  Methanol  Ethyl acetate | *E.coli (ATCC 25922)*  *S.aureus(ATCC 25923)*  *MRSA (ATCC 43300)*  *K.pneumoniae(ATCC 13883)*  *ESBLK.pneumoniae(ATCC 700603)*  *ESBL E. coli, MRSA* | Disc diffusion, Microdilution,FIC | 3 | Multi-drugs | Aqueous and methanolic extracts showed substantial antibacterial activity against *S.aureus*and methicillin-resistant *S.aureus*(MRSA), with MICs ranging from 94µg/ml to 392µg/ml.Italso inhibited the growth of *K. pneumoniae* and *E. coli*, with MIC values of 556µg/ml and 755µg/ml, respectively. The mean and SD values have not been given for the study. |

***Supplementary Table 6. Antibiotic combination effects of* T. chebula *extracts in vitro studies***

***References:***

1. Bag, A., Bhattacharyya, S. K., Bharati, P., Pal, N. K., and Chattopadhyay, R. R. (2009). Evaluation of antibacterial properties of chebulic myrobalan (Terminalia chebula Retz.) extracts against methicillin-resistant Staphylococcus aureus and trimethoprim- sulphamethoxazole-resistant uropathogenicEscherichia coli. Afr. J. Plant Sci. 3 (2), 25–29.
2. Ahuja, C., Kaur, H., and Sharma, R. (2015). Antibacterial activity of Terminalia chebula fruit by agar well diffusion method. J. Chem. Eng. 12, 67–72. doi:10.15415/jce.2015. 12006
3. Gupta, A. (2023). Antibacterial screening of Terminalia chebula Retz. against certain bacterial strains. Environ. Conserv. J. 24 (2), 320–326. doi:10.36953/ECJ.18192546
4. Jinukuti, M. G., and Giri, A. (2013). Antimicrobial activity of aqueous extract of Terminalia chebula Retz. Proteus 30, 0–35.
5. Mostafa, M. G., Mahdia Rahman, M. R., and Karim, M. M. (2011). Antimicrobial activity of Terminalia chebula. Int. J. Med. Arom. Plants 1 (2), 175–179.
6. Prasuna, K., and Devi, Y. P. (2016). Evaluation of Antibacterial Activity of Fruit Extracts of Terminalia Chebulaagainst Human Pathogenic Bacteria. doi:10.5281/zenodo.7350926
7. Naqvi, S. H., Asif, M., Rehman, A. B., and Ahmad, M. (2010). Evaluation of antimicrobial properties of Terminalia chebula Retz. Pak. J. Pharmacol. 27 (1), 29–35.
8. Bag, A., Bhattacharyya, S. K., Pal, N. K., and Chattopadhyay, R. R. (2013). Antibacterial potential of hydroalcoholic extracts of Triphala components against multidrug-resistant uropathogenic bacteria—a preliminary report. Indian J. Exp. Biol. 51 (9), 709–714.
9. Parekh, J., and Chanda, S. (2008). Evaluation of antimicrobial activity of Terminalia chebula Retz. fruit in different solvents. J. Herbs Spices Med. Plants 13 (2), 107–116. doi:10.1300/J044v13n02_10
10. Tariq, A. L., and Reyaz, A. L. (2012). Therapeutic analysis of Terminalia chebula against uropathogenic Escherichia coli (UPEC). Glob. J. Pharmacol. 6 (3), 160–165. doi:10.5829/ idosi.gjp.2012.6.3.64131
11. Selma, H., Rachid, M., Zakia, M., and Fardia, S. (2014). The inhibitory effect of methanolic and aqueous extracts of Terminalia chebula dried fruits on metallo-β- lactamases of Acinetobacter baumannii. Int. J. Pharm. Pharm. Sci. 6, 579–582.
12. Bag, A., Bhattacharyya, S. K., Pal, N. K., and Chattopadhyay, R. R. (2010). Synergistic effect of Terminalia chebulaand antibiotics against multidrug-resistant uropathogenic Escherichia coli. Med. Aromat. Plant Sci. Biotechnol. 5 (1), 70–73.
13. Bag, A., Bhattacharyya, S. K., Pal, N. K., and Chattopadhyay, R. R. (2012). In vitro antimicrobial potential of Terminalia chebula fruit extracts against multidrug-resistant uropathogens. Asian pac. J. Trop. Biomed. 2 (3), S1883–S1887. doi:10.1016/S2221- 1691(12)60514-0
14. Anand Sagar, A. S., and Shaina Parvez, S. P. (2012). Antibacterial activity of Terminalia chebula (Retz.), Emblica officinalis (Gaertn.) and Terminalia bellirica (Gaertn.) Roxb. J. Pure Appl. Microbiol. 6 (3), 1475–1480.
15. Phatthalung, P. N., Chusri, S., and Voravuthikunchai, S. P. (2012). Thai ethnomedicinal plants as resistant modifying agents for combating Acinetobacter baumannii infections. BMC Complement. Altern. Med. 12 (1), 56. doi:10.1186/1472-6882-12-56
16. Singh, D., Singh, D., Choi, S. M., Zo, S. M., Ki, S. B., and Han, S. S. (2012). Therapeutical effect of extracts of Terminalia chebula in inhibiting human pathogens and free radicals. Int. J. Biosci. Biochem. Bioinform 2 (3), 164–167. doi:10.7763/IJBBB.2012.V2.104
17. Tiwana, G., Cock, I. E., and Cheesman, M. J. (2024). Phytochemical analysis and antimicrobial activity of Terminalia bellirica (Gaertn.) Roxb. and Terminalia chebula Retz. fruit extracts against gastrointestinal pathogens: enhancing antibiotic efficacy. Microorganisms 12 (12), 2664. doi:10.3390/microorganisms12122664
18. Singh, G., Kumar, P., and Jindal, A. (2012). Phytochemical study and bioefficacy of Terminalia chebula Retz. against some human pathogens. Int. J. Green Pharm. 6 (4), 289. doi:10.4103/0973-8258.108211
19. Thirunavukkarasu, B., Purushothaman, N., and Valli, S. (2021). Antibacterial activity of Terminalia chebula and Terminalia bellirica fruit extracts against imipenem- and meropenem-resistant Pseudomonas aeruginosa strains. Med. Plants 13 (4), 616–621. doi:10.5958/0975-6892.2021.00071
20. Parveen, R., Shamsi, T. N., Singh, G., Athar, T., and Fatima, S. (2018). Phytochemical analysis and in vitro biochemical characterization of aqueous and methanolic extract of Triphala, a conventional herbal remedy. Biotechnol. Rep. 17, 126–136. doi:10.1016/j.btre. 2018.02.003
21. Khan, I., Ullah, Z., Shad, A. A., Fahim, M., and Öztürk, M. (2022). In vitro antioxidant, anticholinesterase inhibitory, and antimicrobial activity studies of Terminalia chebula (Retz.) and Terminalia arjuna (Roxb.). S. Afr. J. Bot. 146, 395–400. doi:10.1016/j.sajb. 2021.11.016
22. Mishra, M. P., Rath, S., Swain, S. S., Ghosh, G., Das, D., and Padhy, R. N. (2017). In vitro antibacterial activity of crude extracts of nine selected medicinal plants against UTI- causing MDR bacteria. J. King Saud. Univ. Sci. 29 (1), 84–95. doi:10.1016/j.jksus.2015. 05.007
23. Kathirvel, A., and Sujatha, V. (2012). In vitro assessment of antioxidant and antibacterial properties of Terminalia chebula Retz. leaves. Asian pac. J. Trop. Biomed. 2 (2), S788–S795. doi:10.1016/S2221-1691(12)60314-1
24. Bonjar, G. S. (2004). Antibacterial screening of plants used in. Iran. Folkloric Medicine, Fitoterapia 75 (2), 231–235. doi:10.1016/j.fitote.2003.12.013
